# Nuclear medicine imaging for bone metastases assessment: what else besides bone scintigraphy in the era of personalized medicine?

**DOI:** 10.3389/fmed.2023.1320574

**Published:** 2024-01-15

**Authors:** Eric Ouvrard, Ashjan Kaseb, Nathan Poterszman, Clémence Porot, Francois Somme, Alessio Imperiale

**Affiliations:** ^1^Nuclear Medicine and Molecular Imaging, Institut de Cancérologie Strasbourg Europe (ICANS), University Hospitals of Strasbourg, University of Strasbourg, Strasbourg, France; ^2^Radiology, College of Medicine, University of Jeddah, Jeddah, Saudi Arabia; ^3^Radiopharmacy, Institut de Cancérologie Strasbourg Europe (ICANS), Strasbourg, France; ^4^IPHC, UMR 7178, CNRS/Unistra, Strasbourg, France

**Keywords:** bone metastases, bone scan, ^99m^Tc, 18 F-FDG, 18 F-fluorocholine, 68Ga-DOTA, 18F-DOPA, 18F-NaF?

## Abstract

Accurate detection and reliable assessment of therapeutic responses in bone metastases are imperative for guiding treatment decisions, preserving quality of life, and ultimately enhancing overall survival. Nuclear imaging has historically played a pivotal role in this realm, offering a diverse range of radiotracers and imaging modalities. While the conventional bone scan using ^99m^Tc marked bisphosphonates has remained widely utilized, its diagnostic performance is hindered by certain limitations. Positron emission tomography, particularly when coupled with computed tomography, provides improved spatial resolution and diagnostic performance with various pathology-specific radiotracers. This review aims to evaluate the performance of different nuclear imaging modalities in clinical practice for detecting and monitoring the therapeutic responses in bone metastases of diverse origins, addressing their limitations and implications for image interpretation.

## Introduction

The skeleton is the most common organ affected by metastatic spread in solid tumors, notably in breast and prostate carcinomas. Given the prevalence of bone metastasis in specific cancers, vigilant monitoring for bone involvement and prompt intervention are integral aspects of cancer care for individuals at risk. When cancer cells invade the bones, they can disrupt normal bone function, leading to symptoms like bone pain, pathological fractures, and complications related to modifications in bone marrow function. Complications associated with bone metastases are collectively referred to as skeletal-related events (SRE), primarily encompassing pathological fractures, spinal cord compression, and hypercalcemia. SREs are remarkably common among metastatic patients, occurring on average every 3–6 months (1). Early diagnosis and assessment of therapeutic responses to bone metastases are crucial for guiding cancer treatment strategies and minimizing the occurrence of SREs. Managing bone metastasis often involves a range of treatments, including surgery, radiation therapy, bisphosphonate medications, targeted therapies, and palliative care, aimed at alleviating symptoms and enhancing the patient’s quality of life.

In the last decade, significant progress has been made in understanding the tumoral burden on the skeleton. The intricate dependence and interaction between cancer cells and the bone microenvironment has been emphasized. Normal bone tissue undergoes constant dynamic remodeling, where the functions of osteoclastic resorption and osteoblastic production are intricately balanced. Disruption of this delicate equilibrium by cancer cells results in the production of various patterns of bone lesions (2). Skeletal metastases are commonly classified as osteolytic, sclerotic, or osteoblastic based on the radiographic appearance of the lesion. However, schematic dichotomous differentiation is not always possible, as lesions may exhibit both morphological types (mixed metastasis). Predominance of bone resorption mechanisms, generally mediated by activated osteoclasts, leads to the detection of destructive bone lesions, giving metastasis a lytic aspect. Lytic metastases are most common and prevalent in breast, lung, thyroid, renal, and gastro-intestinal malignancies. Myeloma almost always causes osteolytic metastases. Conversely, amplified osteoblastic activity, associated with abnormal formation of frequently unstructured new bone, is prevalent in metastases with a sclerotic appearance. While prostate carcinoma is the main cause of osteoblastic metastases, breast, lung, and neuroendocrine tumors can also cause sclerotic bone lesions.

Metastatic spread predominantly targets skeletal segments rich in highly vascularized red marrow deposits. The physical properties of circulation in these areas, characterized by vascular sinusoids with endothelial cells lacking a basement membrane, facilitate tumor extravasation (3). Consequently, the axial skeleton, ribs, long bones, and vertebral column are commonly affected sites of metastasis. The metastatic process involves a complex multistep interaction between malignant cells and the host microenvironment. Early stages include local tumoral proliferation, cellular detachment, and invasion of organ stroma. Systemic circulation is reached through the penetration of cells into blood vessels and/or lymphatic structures. Adhesion and extravasation into the target organ (bone microenvironment) are crucial stages in metastatic development. Cell adhesion molecules (CAM), expressed on the surface of tumoral cells and in the metastatic site, play a key role in this process, supporting the nonrandom hypothesis of tumor metastases (4, 5). Integrins, a common CAM family, facilitate tumoral cell adhesion to vascular endothelial structures. The rupture of blood vessel basement membranes, induced by the secretion of proteolytic enzymes (i.e., type IV collagenase) by tumoral and host cells, predicts tumoral extravasation through chemotaxis. Subsequent interaction with the microenvironment, involving osteolytic and osteoblastic phenomena, occurs (6). The development of bone metastases can be summarized in four steps (7): (a) bone colonization by circulating tumor cells, formation of a premetastatic niche in the bone marrow, extravasation of circulating tumor cells influenced by pro-migratory and pro-inflammatory tumor-secreted molecules, and tumor cell engraftment; (b) cancer cell dormancy, as the bone marrow environment limits cancer cell proliferation; (c) reactivation of dormant cells after acquiring appropriate genetic mutations allowing them to express bone-specific proteins; (d) disruption of bone homeostasis through the secretion of factors that stimulate or inhibit osteoclast and osteoblast activity, leading to the development of metastases.

## Nuclear medicine imaging

Nuclear medicine imaging has been a cornerstone in the diagnosis and management of bone metastases, playing a vital role in the field of oncology (8, 9). This medical imaging modality provides a diverse array of radiotracers meticulously crafted to address various clinical scenarios. Furthermore, nuclear imaging continues to evolve with technological advancements, making it an essential tool in the detection and monitoring of bone metastases, a common manifestation of advanced cancer. Nuclear medicine enables a non-invasive characterization of tumoral functional status and variability at the molecular and cellular level, examining the uptake intensity and kinetics of several radiotracers. Molecular imaging techniques are highly sensitive, allowing the detection of diseases at the initial stages. To enhance diagnostic accuracy, the integration of anatomical and functional imaging is often achieved through “hybrid” modalities such as Positron Emission Tomography/Computed Tomography (PET/CT) and Single Photon Emission Computed Tomography/Computed Tomography (SPECT/CT) devices (10). While PET/MRI hybrid systems are now available, their role is still being established, and their clinical routine availability is limited.

Understanding the pathophysiology of bone metastases is crucial for comprehending the deployment of various nuclear imaging modalities and their respective strengths and limitations. Nuclear imaging for bone metastasis detection relies on different radiotracers that offer insights into tumor activity, either directly (e.g.,^18^F-fluorodeoxyglucose (^18^F-FDG), ^18^F-Fluorocholine (^18^F-FCH), ^18F-or 68^Ga-radiolabeled prostate-specific membrane antigen (PSMA), ^18^F-Fluoro-dihydroxyphenylalanine (^18^F-DOPA), radiolabeled somatostatin analogs like ^68^Ga-DOTA-peptides) or indirectly, often through the assessment of osteoblastic markers [bisphosphonates labeled with ^99m^Tc, and ^18^F-Fluoride (^18^F-NaF)].

In this narrative review, we will explore the historical significance and the ever-evolving landscape of nuclear imaging in the diagnosis and management of bone metastases, highlighting its pivotal role in improving patient care and outcomes.

## Bone scan: the historical cornerstone in the management of bone metastases

The predominant nuclear imaging technique utilized in the management of bone metastases is bone scintigraphy, commonly referred to as a bone scan (BS). Typically, this involves planar scintigraphy followed by tomographic acquisition, often combined with CT. A ^99m^Tc-labeled bisphosphonate is intravenously administered for this procedure. This radiotracer binds to the mineralization front of hydroxyapatite crystals and the osteocyte gap boundary (11). Consequently, the uptake of the radiotracer is directly influenced by both osteoblastic activity and regional blood flow guiding it to its target.

The BS serves as a valuable tool for estimating the metabolic activity of the skeleton and identifying lesions that cause distinct alterations in physiological bone turnover, even in pre-radiological phases. In contrast, plain radiographs require a 30–50% bone mineral loss before visualizing bone metastases (12). The BS offers the advantage of conducting a whole-body exploration in a short time, with low patient irradiation and high sensitivity for osteoblastic phenomena (13). However, its major drawbacks include a lack of specificity and a low sensitivity for bone lesions with a prevalent osteolytic pattern. Despite the emergence of PET/CT and MRI, the BS still has important indications in oncology, particularly in the work-up of metastatic breast cancer as per the 2021 European Society of Medical Oncology (ESMO) guidelines (14), advanced prostate cancer according to the 2020 American Society of Clinical Oncology guidelines (15), or moderate to high-risk prostate cancer according to the 2020 ESMO guidelines (16). In the following section on the BS, we will focus on these two types of tumors, discussing their performance, limitations, and potential complementarity with other available diagnostic methods.

### Diagnosis of bone lesions

Several studies have conducted a comparative analysis of the diagnostic accuracy between BS and ^18^F-FDG PET/CT in breast cancer. A 2008 meta-analysis revealed a pooled sensitivity for BS of 88% and a specificity of 87% (17). A 2012 systematic review, focusing on newly diagnosed breast cancers, reported a median sensitivity of 98% and a specificity of 93.5% (18). However, within these high median rates, there is variability among studies, with some reporting lower sensitivities. In a more recent 2017 study using histology, clinical, and imaging follow-up as a reference, a sensitivity of 89% was found for BS. Notably, different sensitivities were observed based on the phenotype of metastases, with a sensitivity of 94–100% for osteoblastic and mixed metastases, 90% for osteolytic, and 70% for infraradiologic metastases. In a recent study comparing ^18^F-FDG and BS, a sensitivity of about 51% was found for BS in detecting osteolytic lesions (19). These lower sensitivities are also observed in renal carcinoma, which is mainly responsible for lytic metastases (20–22). Concerning lung cancer, three meta-analyses comparing bone scans to ^18^F-FDG PET with or without CT reported sensitivities and specificities ranging from 86 to 91.8% and 68.8 to 88%, respectively (23–25). All these three meta-analyses concluded to the superiority of ^18^F-FDG imaging.

In the context of prostate cancer, a recent meta-analysis conducted in 2023, comprising 31 studies, compared radiolabeled PSMA PET/CT with conventional imaging for the initial staging of intermediate-to high-risk patients. The meta-analysis reported a pooled sensitivity of 73% and a specificity of 79.1% (26). The sensitivity of BS is impacted by the low detection rate of lytic lesions, which are more common in high-risk patients, and by metastases smaller than the spatial resolution of the gamma camera (even with SPECT acquisitions), both often detected by PSMA PET/CT (26). These variations in sensitivity based on metastasis phenotype should be considered when encountering lytic lesions without significant uptake. Additionally, the effectiveness of BS is limited by their inability to provide specific information about bone lesions, posing challenges in differentiating between benign bone tumors and metastases based solely on uptake patterns. To interpret results accurately and consider the possibility of a bone biopsy, a comprehensive understanding of the distinctive morphological traits of various benign bone tumors and their corresponding uptake levels in a BS is essential (10).

### Follow-up during treatment

Assessing the therapeutic response using bone scintigraphy can be intricate and requires an understanding of various phenomena associated with the evolution of bone metastases in response to treatment (8, 27, 28). Factors such as flare-ups, changes in the density of bone metastases, and potential interferences from bisphosphonates and anti-RANKL agents can complicate the interpretation of results during systemic treatment for patients with bone metastases. These complexities may lead to misleading conclusions about tumor progression or response to the treatment.

#### Flare-up phenomenon

Flare-up is characterized by an apparent progression of bone metastases, such as an increase in the number of bone lesions or enlargement and heightened uptake intensity of known lesions, following the initiation of systemic therapy. This phenomenon is not indicative of actual disease progression. The main mechanisms proposed to explain flare-ups include bone formation replacing necrotic tumoral tissue and inflammatory responses secondary to the destruction of tumor cells. Additionally, in patients with prostate cancer treated with abiraterone, a direct anti-osteolytic and pro-osteoblastic effect of the drug has been observed, leading to increased uptake of bone-targeting radiotracers (29). Flare-up is typically observed in BS imaging but has also been reported in patients undergoing PET/CT with several radiotracers (30–33). This phenomenon is commonly seen within the first 6 months following the introduction of hormonal drugs and chemotherapy in prostate and breast cancer patients (27). It has also been documented in non-small cell lung cancer patients treated with gefitinib between 29 and 77 days after the initiation of treatment (34). To minimize misinterpretation, it is recommended to wait for a period of 6 months after the initiation of systemic treatment before assessing the therapeutic response. In patients with metastatic prostate cancer, the Prostate Cancer Working Group 3 (PCWG3) has established criteria to differentiate between flare-up and real disease progression (35). According to these criteria, a 2 + 2 rule is applied: over the first 24 weeks, patients should undergo a BS every 8–9 weeks. Genuine progression is defined by the identification of at least two new lesions on an initial BS, accompanied by the detection of at least two additional lesions on a subsequent BS.

In the era of SPECT/CT technology, there was optimism that the CT component would provide valuable insights for the interpretation of BS. Unfortunately, it turns out that CT is also susceptible to flare-ups. Among patients with castrate-resistant prostate cancer receiving treatment, a noteworthy 21% experienced an exacerbation of metastatic bone conditions when assessed through a 3 months CT scan, despite observing improvements in their PSA levels. Approximately one-third of these patients did not exhibit any CT progression when re-evaluated 6 months after the start of treatment (36). In addition, the flare-up phenomenon can also be observed during morphological and diffusion MRI sequences, potentially leading to diagnostic errors if not recognized. Finally, despite the challenges in assessing the therapeutic response induced by the flare-up phenomenon, some authors suggest considering it to increase the sensitivity and specificity of bone scintigraphy after treatment initiation, with potential therapeutic impact, particularly on tumors initially staged as localized (37).

#### Decreasing density of bone metastases

**Figure 1:**
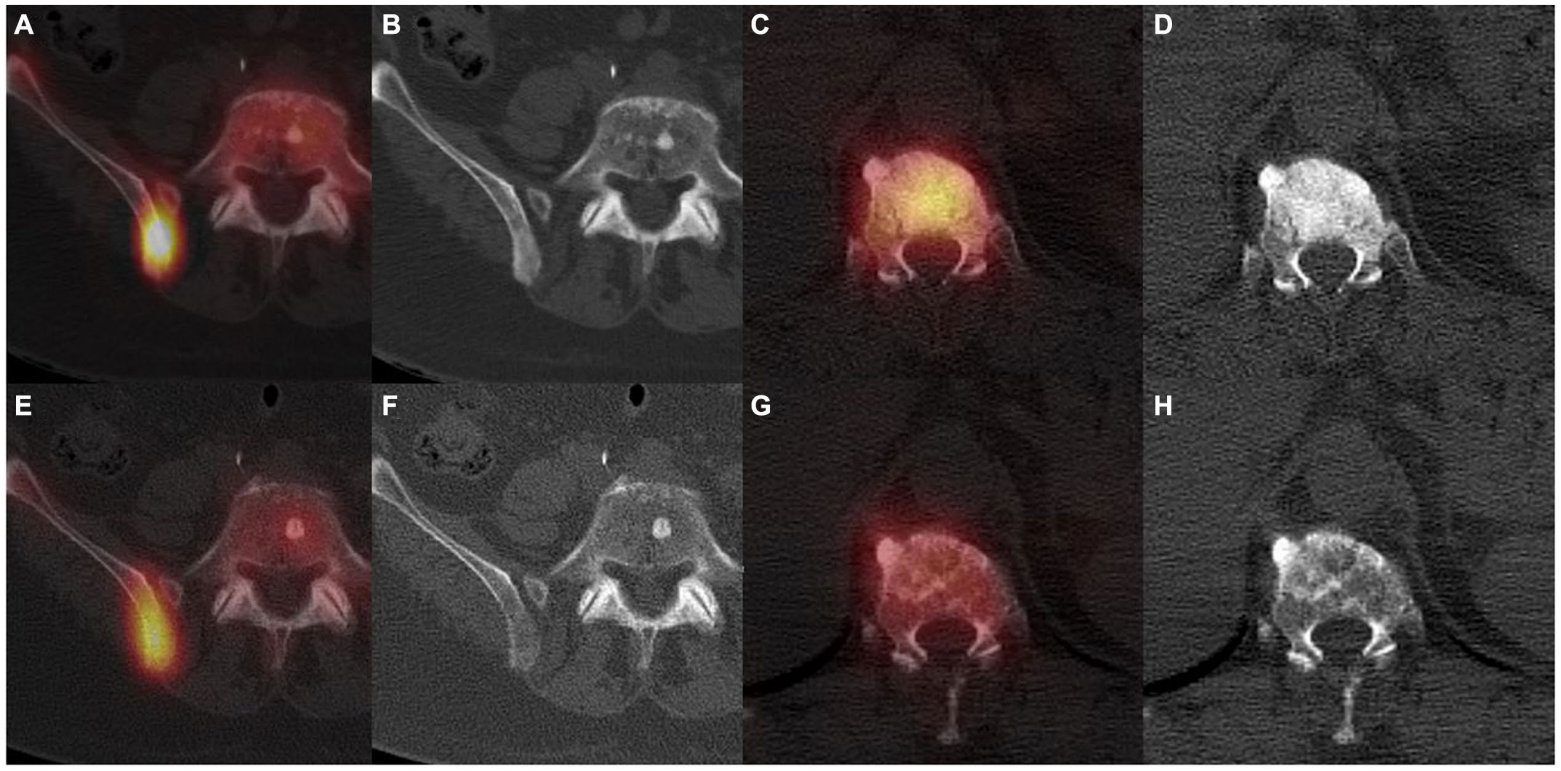
^99m^Tc-HMDP SPECT/CT in a first patient with a bone metastatic prostate adenocarcinoma under first generation hormonotherapy for 2 years, showing a right posterior iliac sclerotic bone metastasis with a high uptake **(A,B)**. Routine follow-up 6 months later showed shading of the iliac metastasis and less intense uptake **(E,F)**, linked to a progression confirmed by a rising PSA and subsequent imaging. ^99m^Tc-HMDP SPECT/CT in a second patient with a bone metastatic breast invasive lobular carcinoma, displaying a vertebral sclerotic metastasis with a high uptake at initial staging **(C,D)**. Routine follow-up 12 months later showed a shadowing of the metastasis and a less intense uptake **(G,H)**, indicative of a therapeutic response confirmed by clinical evolution and subsequent imaging.

Similar to how an increase in the density of sclerotic bone metastases can be associated with either a therapeutic response or progression, studies in prostate cancer under androgen deprivation therapy have found that a decrease in the density of initially sclerotic bone metastases and a reduction in the uptake of bone-targeting agents can also be indicative of both scenarios (Figure 1) (38, 39). This may be attributed to the suppression of the osteoblastic effect of testosterone in the event of a therapeutic response or the progression towards predominant lytic phenomena following the acquisition of resistance to hormonal therapy (38). In prostate cancer, this limitation could be overcome by the advent of radiolabeled PSMA PET/CT. In a retrospective analysis involving prostate cancer patients receiving androgen deprivation therapy, findings revealed that among the 65 patients with bone metastases who underwent ^68^Ga-PSMA PET/CT scans, 15 individuals (23%) displayed a decrease in the density of their bone metastases. Among the 37 lesions that exhibited reduced density, 21 (57%) were negative on PET scans, while 16 (43%) had intense ^68^Ga-PSMA uptake (38). Significantly, PET scans did not detect any positive findings in the bone lesions of patients who exhibited a treatment response.

#### Interference of bisphosphonate and anti-RANKL treatment

The resemblance between bisphosphonates utilized in BS (such as ^99m^Tc-HDP, ^99m^Tc-HMDP, ^99m^Tc-MDP) and those employed for therapeutic applications could give rise to competition for binding sites and reduced tumoral uptake of the radiotracer. This hypothesis was supported by several clinical observations noting a reduced skeletal uptake as soon as 24 h after administration of etidronate (40–43). The persistence of this effect could be observed for a duration of 15 days, as evidenced by consecutive serial BS (42). However, besides small effective sizes, inconsistencies in scanning timing, and the absence of systematic control scintigraphy before bisphosphonate administration, all the reported clinical cases involved etidronate, which is not the main bisphosphonate used in clinical routine.

Three prospective studies addressed this question by comparing bone scintigraphy before and after the administration of different bisphosphonates such as pamidronate, clodronate, zoledronic acid, and alendronate, revealing no notable reduction in bone uptake observed during the course of treatment (44–46). In one autopsy series, the concordance between bone scintigraphy results and bone histology was investigated in 11 patients who had succumbed to androgen-independent prostate cancer. Among them, five had received pamidronate treatment (47). No significant difference in the detection of bone metastases was reported in patients treated with pamidronate.

Today, denosumab, an anti-RANKL that inhibits osteoclast function, has largely replaced bisphosphonate in patients with bone metastases to reduce the risk of SREs. Very few data are available to evaluate its impact on bone scintigraphy. One review reported a case of almost normalization of bone metastases uptake after the introduction of denosumab, despite the rise in biological tumor markers (48). A more recent study compared the uptake intensity of ^18^F-NaF between patients treated by denosumab and a control group using cervical vertebrae as a reference, showing no significant difference (49).

### Response to treatment

The limitations of BS mentioned earlier, along with the gamma camera’s lower spatial resolution compared to PET/CT (approximately 15 mm for SPECT/CT vs. 4 mm for last-generation PET/CT devices), its reduced sensitivity in detecting purely lytic lesions, and the time interval between an effective therapeutic response and the decline in bone-targeting radiotracer uptake, collectively diminish the effectiveness of BS in evaluating treatment responses. Additionally, bone scintigraphy is constrained by moderate interobserver agreement (50).

However, due to their accessibility and relatively low cost compared to PET/CT and whole-body MRI, BS are still widely used in the follow-up of metastatic patients. In this context, caution should be exercised when interpreting changes in BS; confirmation around 8 weeks after the first equivocal BS should be carried out, or further investigations with other imaging modalities may be requested. In the specific case of prostate cancer, the previously mentioned PCWG3 criteria can be applied (35). However, this semi-quantitative method does not account for lesions that are increasing in size and can therefore be misleading for some progressive patients. To address this limitation, an alternative quantitative approach known as the BS Index (BSI), specifically designed for prostate cancer, can be employed (51). It represents the fraction of the total skeleton involved in metastases. However, it remains a time-consuming process and is therefore not easily utilized in clinical routine. An automated version (aBSI) has been developed and is now available. Multiple studies conducted across various clinical scenarios have demonstrated that aBSI plays a prognostic role, exhibiting correlations with overall survival (50).

In the context of breast cancer, MD Anderson criteria can be utilized to assess bone response (52). These criteria combine information from CT, MRI, radiography, and BS to classify the type of response. A prospective study based on MD Anderson criteria found a correlation between tumoral response at 6 months and progression-free survival (53).

In monitoring the treatment response of prostate cancer under ^223^Ra therapy, the pivotal trial did not recommend specific radiological tests (54). Several retrospective studies reported an increase in the number of bone lesions on BS after 12 weeks in 21–28% of patients. However, only very few cases of confirmed progression were observed after six infusions, indicating the possibility of a flare phenomenon (55).

## ^123^I-metaiodobenzylguanidine scintigraphy and bone metastases of neuroblastoma

Neuroblastoma is a common pediatric solid tumor originating from neural crest cells, which give rise to the adrenal medulla and sympathetic nervous system, expressing the norepinephrine transporter (56, 57). More than 50% of neuroblastomas are diagnosed at the metastatic stage (58), often involving bone and bone marrow (59), with only 30–40% long-term survival (60), necessitating more aggressive treatments (61). Therefore, imaging plays a crucial role in the initial staging and response assessment of neuroblastoma to guide therapeutic strategy.

mIBG is a norepinephrine analog taken up by neuroblastoma cells, allowing for the initial staging and response assessment of neuroblastoma, when labelled with ^123^I, scintigraphic imaging. mIBG is excreted into the urinary tract, and its physiologic uptake includes salivary glands, liver, spleen, myocardium, lower to mid-lung zones, colon, brown fat, and uncommonly normal adrenal glands (62). No mIBG physiologic uptake is observed in bone or bone marrow, making it a good tracer to evaluate bone involvement in neuroblastoma (62). Concerning diagnostic performances of ^123^I-mIBG planar scintigraphy in the detection of bone metastases, a 1988 study using histologic data as the gold standard found a sensitivity of 90% and a specificity of 100% (63). The addition of SPECT to planar scintigraphy is recommended, as it allows exact localization of uptake (i.e., bone versus soft-tissue uptake superimposed on planar acquisitions) and improves diagnostic accuracy (62, 64). However, mIBG imaging is time-consuming and suffers from poor spatial resolution compared to other current nuclear medicine modalities. Around 10% of neuroblastoma are not mIBG avid; in these cases, ^18^F-FDG PET/CT is recommended (64). Furthermore, a recent systematic review reported better performances of PET imaging with catecholaminergic tracers than ^123^I-mIBG in lesion-based analysis, notably for bone and bone marrow metastases, thanks to better sensitivity (65).

Several semi-quantitative scores have been developed to measure the extent of the disease and response to treatment. Two of them have shown good inter-observer concordance and a good correlation with outcome (66): SIOPEN score and Curie score, which should be used in initial and follow-up imaging (67). It is worth noting that the SIOPEN score only depends on the extent of bone involvement. More recent recommendations propose using the mIBG relative score (i.e., the absolute score of bone lesions at the time of response assessment divided by the absolute score of bone lesions at baseline before treatment) for response assessment (64).

## Beyond the bone scan: positron emission tomography

### ^18^F-NaF PET, the PET counterpart of BS

^18^F-NaF is a PET bone tracer whose uptake mechanism resembles that of ^99m^Tc-diphosphonates. In this process, ^18^F substitutes for hydroxyl groups in hydroxyapatite and covalently bonds to the surface of new bone. Therefore, the level of ^18^F-NaF uptake depends on both bone formation activity and loco-regional blood flow. Compared to ^99m^Tc-diphosphonates, ^18^F-NaF is characterized by faster pharmacokinetics, allowing image acquisition as soon as 30 min after injection, and a two-fold higher uptake in bone (68). Additionally, it benefits from the better spatial resolution and sensitivity of PET scanners compared to SPECT, providing a theoretically superior capacity to show smaller lesions.

Numerous studies and a few meta-analyses have assessed the diagnostic accuracy of ^18^F-NaF PET/CT in detecting bone metastases from various primary tumors. In a 2019 meta-analysis on a patient-basis, Liu et al. reported various primaries pooled sensitivity of 93% (95% CI, 91–96%) and specificity of 95% (95% CI, 93–96%) when equivocal results were considered as negative, and 96% (95% CI, 93–97%) and 93% (95% CI, 91–55%) when equivocal results were considered as positive. They also included studies comparing ^18^F-NaF PET/CT to planar bone scintigraphy and found that ^18^F-NaF PET/CT shows superior sensitivity and specificity when equivocal results were considered as positive, and superior sensitivity but no significant difference in specificity when equivocal results were considered as negative (69). Concerning prostate cancer specifically, Sheikhbahaei et al. also found excellent diagnostic performance, with a sensitivity of 98% (95% CI, 95–99%) and specificity of 90% (95% CI, 86–93%), surpassing ^99m^Tc-diphosphonate SPECT +/− CT (AUC 0.996 versus 0.896, *p* < 0.001) (70). These results were reproduced in a recent prospective multicenter phase 3 trial comparing ^18^F-NaF PET/CT to ^99m^Tc-MDP SPECT/CT, including patients with breast or prostate cancer and a high risk or a clinical suspicion of bone involvement but without previous documented bone metastasis (71). Based on registry data, Hillner et al. have found that ^18^F-NaF PET, with or without CT, have a significant impact on treatment management of prostate cancer in initial staging and follow-up (72). It should be noted, however, that this study did not make any comparison with bone scan data, making it impossible to compare the impact of the two modalities. This question was partially addressed in a 2019 study, where Zacho et al. found that ^18^F-NaF PET/CT would provide no benefit in patients with a normal bone scan in initial staging (73). In an 81-patient population with moderate to high-risk prostate cancer, ^18^F-NaF indicated bone metastasis in 1 patient and was equivocal in 7 patients, but all of them exhibited biochemical response after radical prostatectomy. On the other hand, a similar study performed in a situation of biochemical relapse after definitive therapy for localized prostate cancer and no relapse found on conventional imaging, including bone scan, found that ^18^F-NaF PET/CT revealed bone metastases in 16% of the population, confirmed by clinical and imaging follow-up (74). However, it is noteworthy that ^18^F-NaF PET/CT found suspicious bone uptake, disproven by follow-up, in 11% of the patients. ^18^F-NaF PET/CT may, therefore, be able to detect bone metastases earlier, as suggested by another study reporting no difference between 18F-NaF PET/CT and BS for the response assessment of bone metastases from prostate cancer, perhaps due to a higher sensitivity at the baseline scan for ^18^F-NaF PET/CT (75).

The quantification of ^18^F-NaF uptake in bone metastases has been utilized in several studies to assess bone response (75–80). SUV_max_ is the main parameter used and has been shown to be correlated with PSA response, alkaline phosphatase kinetics, progression-free survival, and overall survival in prostate cancer (76, 78, 79), and to progression-free survival in breast cancer, using a threshold of ±25% change in SUVmax (81, 82). Similar to BS, ^18^F-NaF PET/CT is also susceptible to the flare phenomenon, as described, for example in breast and prostate cancer (81, 83, 84).

### ^18^F-FDG PET/CT for breast cancer management

^18^F-FDG PET/CT has emerged as a cornerstone in oncology due to its ability to offer valuable insights into tumor metabolic activity. Acting as an analog of glucose, ^18^F-FDG is transported from the blood to metabolically active cells, where it undergoes phosphorylation and, unlike glucose, is not further metabolized. The Warburg effect, which refers to cancer cells’ heightened consumption of glucose compared to normal cells, leads to increased uptake of ^18^F-FDG visible on PET imaging (85). Extensively used in oncology for cancer detection, staging, and restaging, ^18^F-FDG PET/CT enables clinicians to assess the effectiveness of ongoing treatments and identify potential disease recurrence. The ability to visualize metabolic changes before anatomical alterations occur enhances the sensitivity of the technique, contributing to early detection and intervention. Moreover, by gauging changes in metabolic activity post-therapy, clinicians can evaluate the effectiveness of treatments, allowing for timely adjustments to the therapeutic strategy. Various morphological and metabolic criteria have been proposed for this purpose, although they come with limitations in their application to bone metastases and in the context of the advent of targeted therapies and immunotherapy (86). Additionally, ^18^F-FDG PET/CT assists in the localization of biopsy targets, guiding clinicians to areas with heightened metabolic activity for more accurate and informed tissue sampling. Beyond these clinical applications, ^18^F-FDG PET/CT aids in the identification of distant metastases, contributing to a more comprehensive understanding of the disease’s spread. Despite being a powerful tool, ^18^F-FDG PET/CT has limitations, including false positives in areas of inflammation and false negatives in tumors with low glucose metabolism.

#### Initial staging

The shift toward using ^18^F-FDG PET/CT instead of CT and BS in metastatic breast cancer staging (14), as recommended by the 2021 ESMO guidelines, aligns with numerous studies and meta-analyses comparing the diagnostic performances of these modalities. The *per-patient* sensitivities ranged from 81 to 93%, and the specificities ranged from 93 to 99%, with no significant differences noted between ^18^F-FDG PET/CT and BS (17, 87, 88). However, it is worth noting that many patients were already undergoing treatment at the time of imaging, which can complicate definitive interpretations. Responding sclerotic metastases may show persistent radiotracer uptake on BS, even in the absence of ^18^F-FDG uptake. A more recent study involving 84 patients with newly diagnosed metastatic breast cancer compared the performances of ^18^F-FDG PET/CT and BS, revealing that ^18^F-FDG PET/CT detected 87.4% of the total bone lesions, whereas BS identified only 26.3. Moreover, ^18^F-FDG PET/CT led to clinically relevant management differences in 16% of patients compared to BS (89).

**Figure 2:**
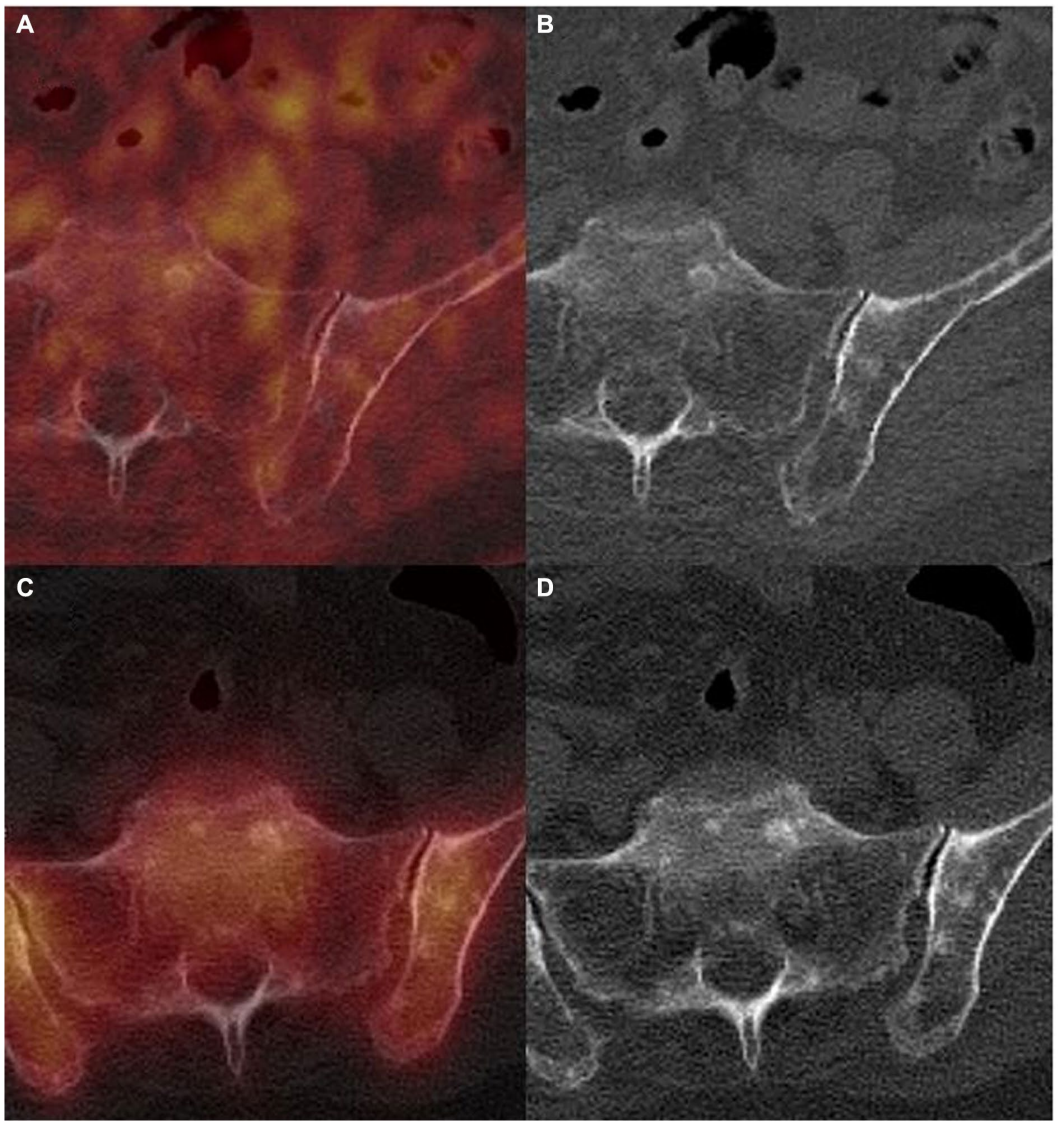
^18^F-FDG PET/CT **(A,B)** and ^99m^Tc-HMDP SPECT/CT **(C,D)** in a 75 years-old woman referred for initial staging of an invasive lobular carcinoma of the breast, showing multiples subcentimetric sclerotic bone lesions, linked to a diffuse bone invasion on the rest of the scans (not shown), without ^18^F-FDG and ^99m^Tc-HMDP uptake, corresponding to bone metastases confirmed by biopsy.

The immunohistochemical characteristics of breast cancers, such as estrogen receptor (ER), progesterone receptor (PR), HER2 receptor expression, and Ki67, play a crucial role in determining therapeutic options and are also correlated with tumor aggressiveness. This correlation extends to the degree of ^18^F-FDG uptake, as confirmed by a recent meta-analysis. Tumors that are ER-negative, PR-negative, HER2-positive, and have a high Ki67 (>14%) exhibit significantly increased SUVmax (90). The histologic subtype of breast cancer further influences the level of ^18^F-FDG uptake, with invasive ductal carcinoma (IDC) being more ^18^F-FDG-avid than invasive lobular carcinoma (ILC) (91–93). Additionally, ILC bone metastases tend to be more often sclerotic than IDC metastases (91). Interestingly, BS appears to outperform ^18^F-FDG PET/CT in detecting sclerotic metastases, with detection rates of 55.6–74% versus 95–100% for bone scintigraphy (Figure 2) (94, 95).

As previously indicated, ^18^F-FDG PET/CT outperforms BS in the initial assessment not only of non-small cell lung carcinoma (23–25) but also in a variety of primitive such as renal carcinoma (96), hepatocellular carcinoma (97), head and neck cancers (98), or osteosarcoma (99).

In the pre-therapeutic context, it is crucial to consider the histological subtype and immunohistochemical characteristics when encountering a consolidating bone lesion without ^18^F-FDG uptake. For tumors with low ^18^F-FDG avidity, the likelihood of a bone metastasis remains significant. It becomes important in such cases to search for other lesions with a similar phenotype that may exhibit uptake and to assess for morphologic progression based on previous imaging studies.

#### Response assessment

The efficacy of ^18^F-FDG PET/CT in evaluating therapeutic bone responses has been investigated in studies involving patients with bone-only or bone-dominant metastatic breast cancer. There is a correlation between the evolution of tumoral uptake and the response assessed by changes in biological tumor markers, conventional imaging, and subjective symptoms in bone-dominant metastatic breast cancer (100). Furthermore, a retrospective study with 102 patients demonstrated that a decrease in ^18^F-FDG uptake was an independent predictor of a longer response duration to treatment (101). These findings have been substantiated in recent studies (102, 103), indicating a significant correlation between higher uptake and shorter progression-free survival and shorter time before SRE occurs. Additionally, ^18^F-FDG PET/CT appears capable of detecting a quantifiable measurable response earlier than CT in retrospective a series (104). In a prospective study, including 26% of patients with bone-only metastatic breast cancer, ^18^F-FDG PET/CT identified disease progression 6 months earlier than CT did (105). Hence, ^18^F-FDG PET/CT emerges as a reliable tool for assessing bone response to treatment, potentially influencing clinical therapeutic decisions compared to BS and CT. It is worth noting that a flare-up phenomenon has been reported within the first 7–10 days following the introduction of tamoxifen in a prospective trial evaluating the role of ^18^F-FDG PET/CT in tamoxifen response assessment in breast cancer (106). However, this effect does not seem to persist over time, making it unlikely to be a source of misinterpretation. In the case of non-small cell lung cancer, a flare phenomenon has also been described in a series of four cases after 2 to 3 cycles (6 to 9 weeks) of bevacizumab associated with standard chemotherapy (107). It is worth noting that this effect did not involve non-osseous lesions.

### Radiolabeled choline and PSMA PET/CT for prostate cancer management

Prostate cancer cells usually exhibit low avidity for ^18^F-FDG, often showing no uptake on PET/CT scans. To address this limitation, two main radiotracers are available for molecular imaging of prostate cancer: radiolabeled choline and PSMA ligand (108, 109). Choline derivatives labeled with ^18^F or ^11^C are incorporated into phosphatidylcholine, a constituent of cell membranes, whose metabolism is increased in prostate cancer cells. PSMA, an enzyme highly expressed at the membranes of prostate cancer cells, serves as a target for radiolabeled PSMA ligands. These ligands bind to this enzyme and are subsequently internalized by the cell. Beyond its theragnostic potential via ^177^-Lutetium (^177^Lu)-PSMA, it has been demonstrated that this radiotracer is even more specific than radiolabeled choline, enabling the detection of millimetric or infraradiologic bone lesions.

#### Initial staging

Considering T-stage and N-stage, the less detailed anatomical information from CT (compared to MRI), the influence of the partial-volume effect, and the limited sensitivity for the detection of micro metastases appear to be the most significant drawbacks for accurate staging with choline PET/CT (108). Various studies have assessed ^18^F-FCh PET/CT in the M staging of medium-to high-risk prostate cancer. Langsteger et al. reported a sensitivity and specificity of 91 and 83%, respectively, for the detection of bone metastases in prostate cancer patients (110). Wondergem et al. confirmed these favorable performances, reporting on a patient basis that sensitivity and specificity rates were 85.2 and 96.5% for ^11^C-Choline and ^18^F-FCh (111). It appears that ^18^F-FCh PET/CT is more sensitive in the early phase of the bone metastatic process, when the lesions are in the bone marrow and no cortical lesions are evident. In the clinical phase of the process, ^18^F-FCh PET/CT and bone scintigraphy have similar diagnostic performances.

**Figure 3:**
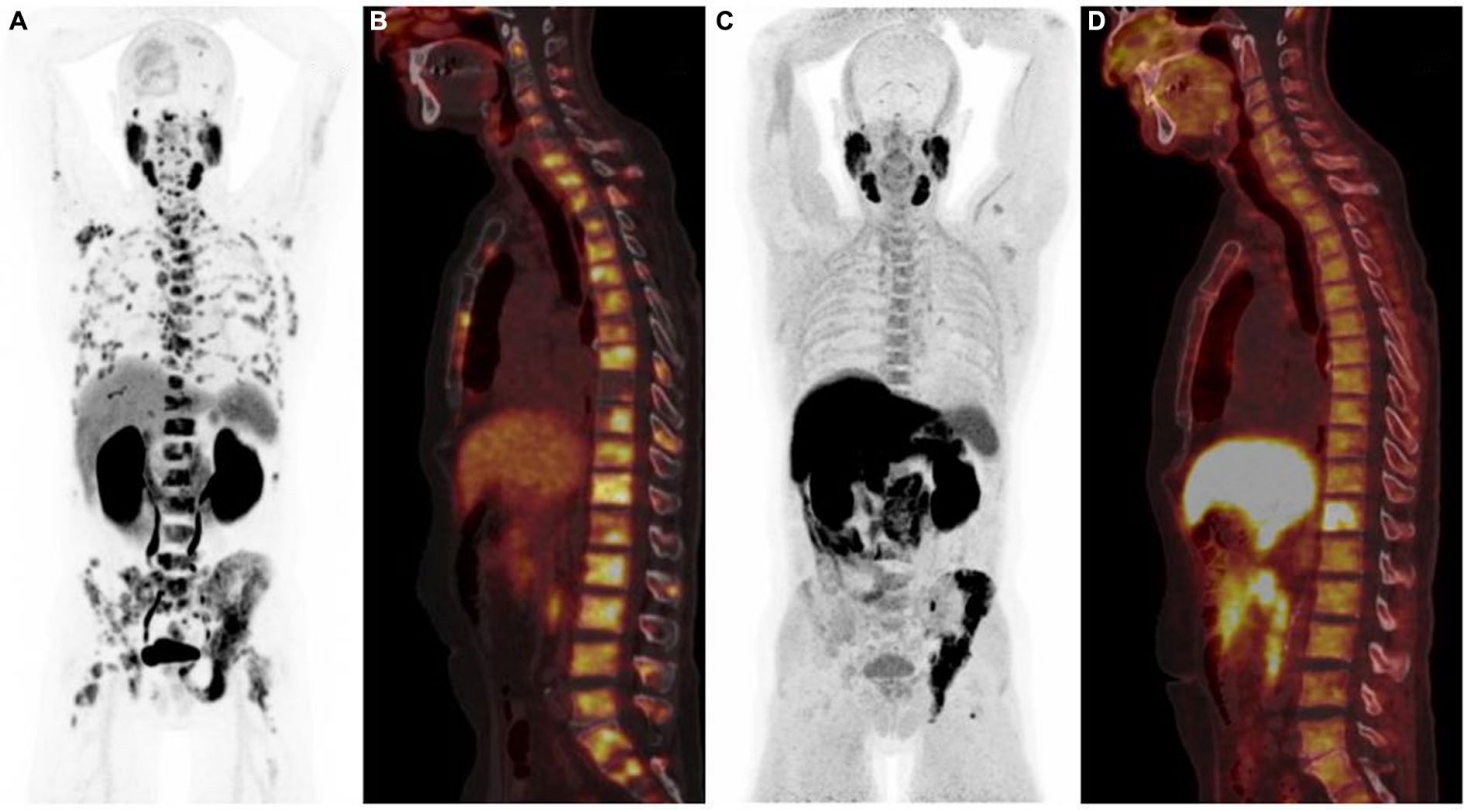
^68^Ga-PSMA PET/CT [anterior MIP **(A)**, sagittal PET/CT **(B)**] and ^18^F-Fluorocholine PET/CT [anterior MIP **(C)**, sagittal PET/CT **(D)**] illustrating the difference in sensitivity between the two radiotracers, ^68^Ga-PSMA PET/CT showing more bone metastases than ^18^F-Fluorocholine scan.

PSMA PET/CT also demonstrates excellent performance in the initial M staging of prostate cancer (Figure 3). An intriguing retrospective study involving 30 patients assessed the uptake of ^68^Ga-PSMA-11 and the type of bone metastases (112). Surprisingly, the study revealed that radiotracer uptake was significantly higher in osteolytic and bone marrow metastases compared to osteoblastic ones. Among 126 patients who underwent PSMA PET and planar BS within 3 months, 37 patients were in the case of initial staging (113). In a patient-based analysis, the sensitivity and specificity were 100% (IC 95 76.8–100%) versus 71.4% (IC 95 41.9–91.6%), and 100% (IC 95 85.2–100%) versus 95.7% (IC 95 78.1–99.9%; *p* = 0.006) respectively. Hofman et al. recently published a prospective, randomized, multicenter study called proPSMA (114). Three hundred and two patients with high-risk prostate cancer were randomly assigned to conventional imaging (i.e., bone scintigraphy and computed tomography) or PSMA PET/CT for the initial staging. PSMA PET-CT had a 27% (95% IC 23–31%) greater accuracy than that of conventional imaging: 92% (95% IC 88–95%) versus 65% (95% IC 60–69%); *p* < 0.0001. Bone metastases were detected in 15/150 (10%) patients in the PSMA PET/CT group. Most interestingly, first-line conventional imaging conferred management change less frequently: 23% (95% IC 10–22%) versus 41% (95% IC 21–36%; *p* = 0.008).

Finally, one should be aware of the high rate of false-positive focal bone uptake with PSMA PET/CT, known as unspecific bone uptake (also known as UBU), whose etiology is still unknown. Several studies have reported a higher incidence with ^18^F-F-PSMA than with ^68^Ga-PSMA (115). Comparison with CT, other prostate-specific radiotracers, clinical history, and previous traumatic events is recommended to overcome this limitation (115).

#### Biochemical recurrence after a curative intent treatment

This stage of the disease is the most extensively studied for both choline and PSMA PET/CT. Fanti et al. published a meta-analysis of 18 articles with 2,126 patients evaluating the detection rate of ^11^C-Choline PET/CT (116). The pooled detection rate, sensitivity, and specificity were 62% (95% IC 53–71%), 89% (95% IC 83–93%), and 89% (95% IC 73–96%) respectively. For bone metastases, eight studies with 775 participants reported detection rates. The pooled rate was 25% (95% IC 16–34%). Another meta-analysis focused on the detection of bone metastasis. Guo et al. analyzed 14 studies of 840 patients (117). On a per-patient analysis, the authors reported a pooled sensitivity, specificity, and negative likelihood ratio of 89% (95% CI 80–94%), 98% (95% CI 95–99%), and 12% (95% CI 7–20%) respectively. It is worth mentioning that despite its excellent performance for the detection of bone metastasis, a negative choline PET/CT cannot rule out the absence of bone lesions.

PSMA PET/CT has outperformed choline PET/CT in the diagnosis of biochemical relapse. A head-to-head comparison of ^68^Ga-PSMA and ^18^F-choline PET/CT was conducted on 37 patients (118). The detection rates were 86.5 and 70.3%, respectively, in favor of the PSMA PET/CT (*p* = 0.04). Schwenck et al. confirmed the superiority of PSMA PET/CT (versus ^11^C-choline PET/CT) for the detection of both lymph nodes (94% versus 71%, *p* < 0.001) and bone metastases (98% versus 64%, *p* < 0.001) in this clinical setting (119). Moreover, it appears that the real benefit of PSMA PET/CT is in biological recurrences with low PSA levels. Burgard et al. retrospectively analyzed 115 patients with PSA levels under 0.2 ng/mL (120). Overall, 29 patients (25.2%) had lesions suspicious of prostate cancer. Eleven (25%) lesions out of 44 were bone metastases. Another recent meta-analysis confirmed these data (121). Thirty-seven articles with 4,790 patients were included. The authors reported that for PSA categories 0–0.19, 0.2–0.49, 0.5–0.99, 1–1.99, and ≥2 ng/mL, the percentages of positive scans were 33, 45, 59, 75, and 95%, respectively.

Of note, the performance of radiolabeled-choline PET/CT depends on both the PSA value at the time of the examination and the PSA doubling time (PSAdt). Castelluci et al. evaluated 605 patients treated with radical prostatectomy with an early biochemical relapse (PSA values between 0.2 ng/mL and 2 ng/mL; mean 1.05 ng/mL; median 1.07 ng/mL) (122). They reported an overall detection rate of 28.4% (172/605 patients). Bone lesions were observed in 56 patients (9.3%). The multivariate analysis confirmed that PSA value (*p* = 0.011) and PSA doubling time (*p* < 0.001) were significant predictors of PET/CT positivity, with optimal cutoff values of 1.05 ng/mL and 5.95 months. This also concerns the ligand PET/CT. A retrospective analysis of 1,007 patients who underwent ^68^Ga-PSMA PET/CT reported an overall detection rate of 79.5% (801 patients) (123), with 131 (13%) patients diagnosed with bone metastases. The authors confirmed that PSA level was significantly associated with a pathological PET/CT result (*p* < 0.001).

#### Therapy response assessment

A very few studies have evaluated choline PET/CT for the therapy response assessment of castration-resistant prostate cancer, whether with chemotherapy or hormone therapy. Schwarzenböck et al. monitored 11 patients treated with docetaxel (124), De Giorgi et al. evaluated 43 patients under abiraterone (69), and 36 patients under enzalutamide (70). These studies reported a high percentage of discrepancy between PSA and PET/CT results and did not provide many details on the evolution of bone metastases under treatment.

On the other hand, there is rapidly growing evidence that promises a place for PSMA PET/CT in this indication. The first step was to create an international framework for response evaluation criteria. The question is not fully resolved yet, with several classifications already in use: the adapted PCWG3 (using PSMA PET instead of bone scan), the PSMA PET Progression criteria (PPP) (125), and the RECIP 1.0 classification (126). Gafita et al. compared all those criteria in 124 men with a metastatic RCPC (127) and demonstrated that the RECIP 1.0 classification was the most correlated to patient outcomes.

As already explained, PCWG3 defines bone progression as the appearance of at least 2 new lesions (with two or more additional new lesions on a confirmatory scan, 2 + 2 rule). In contrast, because of its high specificity, 1 new distant lesion by PSMA PET is considered progressive disease by PPP if the following criteria are met: consistent clinical or laboratory data, including PSA and other parameters such as pain assessment, lactate dehydrogenase, and anemia (125). The RECIP 1.0 criteria mainly focus on the PSMA uptake volume for therapy response assessment under ^177^Lu-PSMA therapy (126). The definition of a new lesion is as follows: the appearance of at least 1 new PSMA-positive lesion, defined as any new focal uptake of PSMA ligand higher than the surrounding background. The authors recognized that there was no external validation of their threshold definition and that they could not compare the prognostic ability of RECIP versus PCWG3 criteria, since bone scans were not included in the clinical workup of ^177^Lu-PSMA radionuclide therapy at all institutions.

One prospective trial studied the role of PSMA PET/CT in the follow-up of ^223^Ra therapy and showed progressive bone disease in 89% of the patients, with the overall burden of disease on PSMA being strongly correlated with PSA changes (128). Since PSA seems inappropriate for evaluating the response to ^223^Ra therapy (128, 129), the role of PSMA PET/CT for monitoring patients under ^223^Ra appears limited.

Focusing on bone metastases, Schmidkonz et al. investigated 177 men suffering from 443 bone lesions on ^68^Ga-PSMA-11 PET (130). Within this cohort, 20 patients with 173 bone metastases underwent PSMA PET/CT imaging before and after therapies. Bone metastases showed a mean density of 589 ± 203 HU before therapy, with a significant increase to 827 ± 215 HU (*p* < 0.05). None of the other CT-derived parameters had significant changes under therapies. In contrast, a significant correlation was observed between the percentage differences of whole-body total metabolic volume and the percentage difference of serum-PSA levels (*p* < 0.001) before and after therapies. The main limitation was the vast heterogeneity of treatments used (radiation therapy, androgen deprivation therapy, chemotherapy, and radioligand therapy).

The flare phenomenon has also been described in several case reports and studies between 2 and 6 weeks following treatment initiation, depending on study design (131–133). It is not exactly known how long a flare-up can be observed on PSMA PET/CT, but it is worth noting that one study described no flare phenomenon after a median follow-up of 3 months in 26 patients (134).

### ^18^F-FDOPA and ^68^Ga-DOTA-peptides PET/CT for neuroendocrine tumors management

Neuroendocrine tumors (NETs) are rare and heterogeneous epithelial neoplasms with neuroendocrine differentiation. The primary site of origin for NETs, accounting for approximately 60% of cases, is the gastrointestinal (GI) tract, with the small intestine being the foremost location of tumor development (135, 136). The incidence of bone metastases in patients with NETs has been investigated mainly through retrospective studies. Data from US institutional registries indicate that around 12% of patients with NETs develop bone metastatic disease (137, 138). This finding aligns with the results of a recent European study, which examined 677 patients diagnosed with NETs from 2000 to 2015 (139). Generally, bone metastases in NETs appear to be more frequent in lung carcinoids compared to gastroenteropancreatic neuroendocrine tumors (GEP-NETs). Among GEP-NETs, rectal NETs consistently exhibit the highest propensity for metastasizing to the bones (140).

While bone involvement is incidentally discovered in as many as 40% of NET patients, clinical complications arising from bone metastases have been noted in roughly half of these cases (138). Typically, NET bone metastases affect the axial skeleton more frequently than the appendicular bones, and identifying radiographic signs can be challenging. When standard radiography and CT scans are employed, NET bone metastases tend to exhibit either an osteosclerotic pattern or a combination of osteolytic and osteosclerotic features. It seems that MRI exhibits higher sensitivity in the detection of NET bone lesions compared to CT. Whole-body MRI with diffusion-weighted imaging, in particular, is noted for its substantial diagnostic accuracy in this regard (141–144).

The prognosis for NET patients with bone metastases varies widely based on factors such as the extent of bone involvement, the aggressiveness of the primary tumor, and the effectiveness of treatment. While bone metastases in NETs are generally associated with a more indolent course compared to some other cancers, they can still significantly impact a patient’s quality of life and overall survival. Evidence indicates that NET patients diagnosed with synchronous bone metastases tend to experience less favorable survival outcomes when compared to those with metachronous bone metastases that appear later after diagnosis (145, 146). Interestingly, the impact of bone metastases on survival appears to be more significant in patients with pancreatic NETs than with intestinal NETs (147). Notably, in lung NETs, the presence of bone metastases is associated with a poor prognosis, regardless of the tumor grade or whether the metastases occur synchronously or metachronously (141).

Over the last 20 years, nuclear medicine imaging methods for NETs have undergone gradual advancements (148, 149). The introduction of radiotracers such as ^18^F-Fluoro-dihydroxyphenylalanine (^18^F-DOPA) and ^68^Ga-DOTA-peptides for PET imaging has revolutionized the diagnosis and management of NETs. These cutting-edge tools provide unprecedented insights into localization, characterization, and treatment planning, elevating the precision of care for patients grappling with these complex malignancies (150).

**Figure 4:**
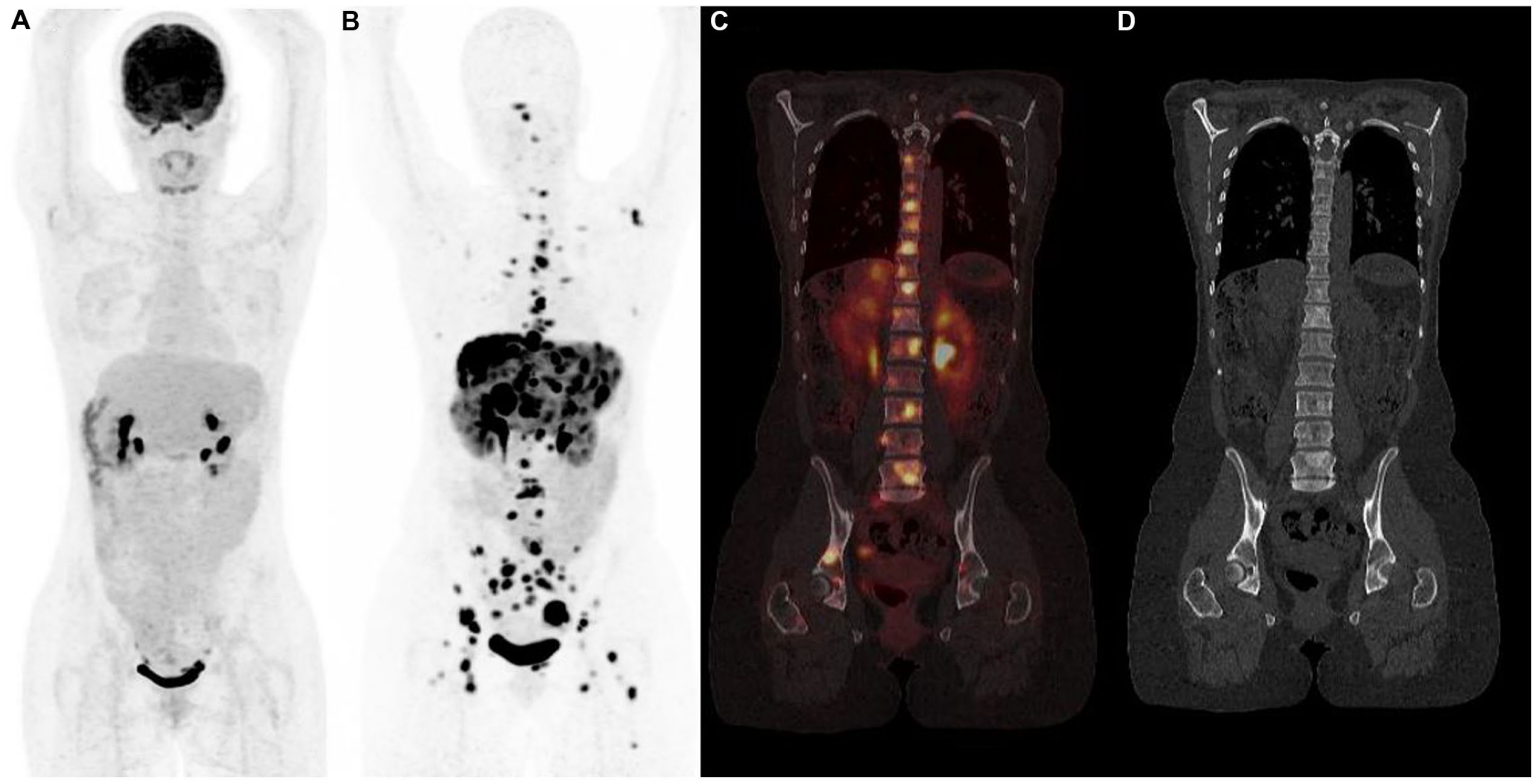
^18^F-FDG **(A)** and ^68^Ga-DOTATOC **(B–D)** PET/CT results (anterior MIP, coronal PET/CT, coronal CT) in a 48 years-old woman with grade 2 metastatic pancreatic neuroendocrine tumor showing multiple bone sclerotic metastases characterized by intense and pathologic ^68^Ga-DOTATOC uptake. ^18^F-FDG PET/CT failed to detect metastatic spread.

Due to the moderate-to-high overexpression of somatostatin receptors (SSTR), predominantly subtype 2A, the primary imaging technique for diagnosis and assessment involves SSTR imaging. Synthetic somatostatin analogs (SSAs), such as octreotide, have been effectively labeled for medical imaging. Among these radiopharmaceuticals, ^111^In-pentetreotide (OctreoScan) has demonstrated its utility for gamma camera studies over more than two decades. Recently, a new category of somatostatin analogs, labeled with the positron-emitting radionuclide ^68^Ga for PET/CT imaging and called ^68^Ga-DOTA-peptides (^68^Ga-DOTATOC, ^68^Ga-DOTATATE, and ^68^Ga-DOTANOC), has emerged as the current gold standard for assessing NETs (148, 149). The introduction of ^68^Ga-DOTA-peptide PET/CT has significantly improved the detection of skeletal metastases, surpassing both bone scintigraphy and OctreoScan scintigraphy in performance. Recent studies indicate that ^68^Ga-DOTA-peptide PET/CT demonstrates a diagnostic accuracy ranging from 80 to 100% in identifying NET bone lesions (151–154). When compared to contrast-enhanced CT and MRI, ^68^Ga-DOTA-peptide PET/CT may enhance diagnostic capabilities by approximately 20 to 25% in the detection of bone metastases (Figure 4) (151, 152, 155, 156).

Peptide Receptor Radionuclide Therapy (PRRT) is a targeted therapeutic approach for NETs involving the administration of a radiolabeled somatostatin analog, typically ^177^Lu. This analog specifically binds to somatostatin receptors overexpressed in NETs, leading to the selective delivery of beta particle-emitting radiation and localized cytotoxic effects. PRRT has demonstrated efficacy in controlling tumor progression, especially in metastatic scenarios where conventional treatment modalities may be limited (157). As a personalized and targeted therapeutic strategy, PRRT holds promise in optimizing outcomes and managing symptoms associated with NETs. Post-treatment SPECT/CT imaging after PRRT is critical for dosimetric evaluation (158) and is potentially useful for monitoring treatment efficacy and promptly detecting disease progression during treatment. However, no studies have specifically focused on the efficacy of post-PRRT SPECT/CT imaging for bone metastases monitoring. On the other hand, ^68^Ga-DOTA-peptide PET/CT has been successfully used to monitor PRRT efficacy in patients with bone metastases of NETs treated by ^177^Lu-DOTA-octreotate. The treatment results in prolonged overall survival and provides relief from pain, supporting the consideration of PRRT in the management of advanced bone metastatic disease (159, 160).

Importantly, non-tumoral processes with elevated osteoblastic activity can also present challenges in the interpretation of ^68^Ga-DOTA-peptides PET, as they may exhibit increased osseous uptake. These situations encompass a range of conditions, including degenerative alterations, fractures, and non-malignant lesions like hemangioma, enchondroma, and fibrous dysplasia. Additionally, meningiomas, often incidentally discovered, manifest as intensely avid masses located outside the brain parenchyma. They are typically found along the cerebral convexity, in the parasagittal region, or originating from the sphenoid wing (161).

In addition to ^68^Ga-DOTA-peptides, ^18^F-DOPA has been successfully proposed for *in vivo* nuclear imaging of NETs (162, 163). The heightened uptake of ^18^F-DOPA in NETs results from increased cellular synthesis, storage, and secretion of biogenic amines. As a result, the sensitivity of ^18^F-DOPA PET is influenced by the NET’s embryological origin and differentiation. Specific tumoral features, such as the biosynthesis of serotonin, play a significant role in explaining the superior sensitivity of ^18^F-DOPA PET for small intestine carcinoids (SiNET). ^18^F-FDOPA PET outperforms morphological imaging (CT) and gamma camera-based SSTR imaging for the detection of skeletal metastases, lymph nodes, and liver lesions in patients with low-grade midgut NETs. The sensitivity of ^18^F-FDOPA PET was 100, 95, and 96% respectively, in per patient, per region, and per lesion analysis (164). Becherer et al. reported sensitivities of 90.9% for the skeleton in the evaluation of patients with histologically proven NETs (165). In a recent study by Deleval et al., ^18^F-DOPA PET/CT detected bone metastases in 46 of 155 (29.7%) SiNET patients, with a negative prognostic impact (166). In a recent ^18^F-DOPA PET/CT study, Lelièvre et al. (167) described the topographical distribution of bone metastases in patients with SiNET, mainly involving the spine, pelvic bones, and ribs. Metabolic tumor volume (MTV), excluding bone lesions, greater than 19.2 mL, and hepatic metastatic involvement were significant predictors of bone metastases.

More recently, several retrospective studies (168–171) and one systematic review (172) have compared ^18^F-FDOPA PET/CT and SSTR PET/CT in well-differentiated small intestine NETs. Despite similar high patient-and region-based pooled sensitivities (83 and 89%, respectively, for ^18^F-DOPA PET; 88 and 92%, respectively, for SSTR PET), ^18^F-DOPA demonstrated superiority in lesion detection (lesion-based pooled sensitivity, 95% vs. 82%). This higher diagnostic performance was also observed in the case of bone metastases (168, 169). Specifically, ^18^F-DOPA PET/CT exhibited greater sensitivity than ^68^Ga-DOTA peptides PET/CT in patients with high levels of serotonin and 5-hydroxyindoleacetic acid (169, 170).

**Figure 5:**
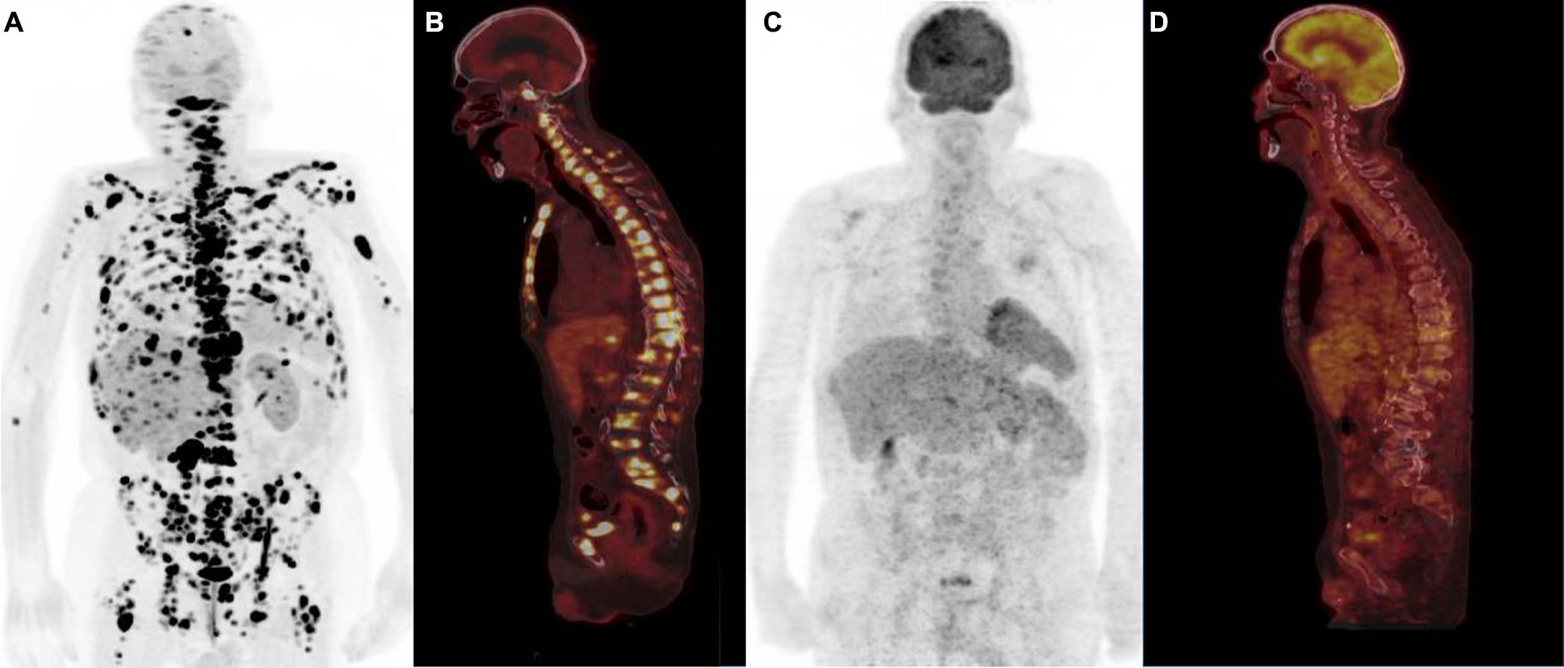
Typical example of “flip-flop” effect (anterior PET MIP, sagittal PET/CT) in a patient with metastatic low-grade (G1) small bowel NET. 18F-FDOPA PET showed multiple sclerotic bone metastases **(A,B)** not detectable by 18F-FDG PET **(C,D)**, emphasizing the role of tumor grade in the selection of the optimal diagnostic radiotracer.

The limited value of ^18^F-FDG PET/CT is typically reported in the management of low-grade NETs, likely due to their low metabolic activity and slow growth (Figure 5). Despite these considerations, ^18^F-FDG PET holds potential for prognostic stratification in patients with NETs. Notably, NETs exhibiting increased ^18^F-FDG uptake tend to display more aggressive behavior, leading to a less favorable long-term prognosis (173).

## Conclusion

Despite their widespread use in staging and assessing bone metastases, BS have limitations impacting sensitivity and specificity, particularly in the follow-up of patients undergoing therapy. PET/CT has effectively addressed these limitations, demonstrating potential therapeutic impact, as seen in breast cancer. Nuclear bone imaging is now evolving towards the use of more specific PET tracers tailored to each tumor type, a trend already established for prostate and neuroendocrine tumors. Additionally, the advent of large field-of-view PET/CT, enabling dynamic imaging of extensive portions of the body, should have an interest in response assessment of bone metastases. Finally, PET/MR devices may contribute to enhancing diagnostic performance by leveraging complementary information from both modalities.

## Author contributions

EO: Supervision, Validation, Writing – original draft, Writing – review & editing. AK: Writing – review & editing. NP: Writing – review & editing. CP: Writing – review & editing. FS: Writing – review & editing, Writing – original draft. AI: Writing – original draft, Writing – review & editing.
